# Measurement of the spin-forbidden dark excitons in MoS_2_ and MoSe_2_ monolayers

**DOI:** 10.1038/s41467-020-17608-4

**Published:** 2020-08-12

**Authors:** C. Robert, B. Han, P. Kapuscinski, A. Delhomme, C. Faugeras, T. Amand, M. R. Molas, M. Bartos, K. Watanabe, T. Taniguchi, B. Urbaszek, M. Potemski, X. Marie

**Affiliations:** 1 University of Toulouse, INSA-CNRS-UPS, LPCNO, 135 Av. Rangueil, 31077 Toulouse, France; 2 Laboratoire National des Champs Magnétiques Intenses, CNRS-UGA-UPS-INSA-EMFL, 38042 Grenoble, France; 3 Department of Experimental Physics, Faculty of Fundamental Problems of Technology, Wrocław University of Science and Technology, Wybrzeże Wyspiańskiego 27, 50-370 Wrocław, Poland; 4 Institute of Experimental Physics, Faculty of Physics, University of Warsaw, ul. Pasteura 5, 02-093 Warsaw, Poland; 5 Central European Institute of Technology, Brno University of Technology, Purkynova 656/123, 61200 Brno, Czech Republic; 6 National Institute for Materials Science, Tsukuba, Ibaraki 305-0044 Japan

**Keywords:** Materials science, Physics

## Abstract

Excitons with binding energies of a few hundreds of meV control the optical properties of transition metal dichalcogenide monolayers. Knowledge of the fine structure of these excitons is therefore essential to understand the optoelectronic properties of these 2D materials. Here we measure the exciton fine structure of MoS_2_ and MoSe_2_ monolayers encapsulated in boron nitride by magneto-photoluminescence spectroscopy in magnetic fields up to 30 T. The experiments performed in transverse magnetic field reveal a brightening of the spin-forbidden dark excitons in MoS_2_ monolayer: we find that the dark excitons appear at 14 meV below the bright ones. Measurements performed in tilted magnetic field provide a conceivable description of the neutral exciton fine structure. The experimental results are in agreement with a model taking into account the effect of the exchange interaction on both the bright and dark exciton states as well as the interaction with the magnetic field.

## Abstract (web summary)

Excitons control the optical properties of transition metal dichalcogenide monolayers. Here, the authors measure the exciton fine structure of MoS_2_ and MoSe_2_ monolayers encapsulated in hBN in magnetic fields up to 30 T, and observe a brightening of the spin-forbidden dark excitons in MoS_2_.

## Introduction

Transition Metal Dichalcogenide (TMD) monolayers such as MoS_2_, MoSe_2_, WS_2_ or WSe_2_ are 2D semiconductors characterized by very strong interaction with light due to robust excitons with large oscillator strengths^1–5^. As for all semiconductor nanostructures the exciton fine structure dictates the efficiency of the coupling to light. One can expect that the optoelectronic properties will change drastically whether the spin-forbidden dark excitons lie below or above the bright excitons^6–9^. Exciton spin relaxation is also expected to be affected by the bright-dark exciton ordering^10,11^.

The main difficulty to experimentally determine the energy of spin-forbidden dark excitons is their extremely small oscillator strength compared to the bright exciton one. The exciton fine structure splitting was accurately determined for WS_2_ and WSe_2_ monolayers (ML) using various experimental techniques^12–15^. These measurements were successful only in WSe_2_ and WS_2_ materials as the dark exciton lie several tens of meV below the bright one so that the small oscillator strength is compensated by a very large population of dark excitons making them observable in photoluminescence experiments. In contrast the respective alignment of bright and dark excitons in MoS_2_ ML remains controversial though this material is the most studied among the 2D semiconductors and this was the first member of the TMD family to be established as a direct gap in the monolayer form.

Numerous ab-initio calculations have been proposed to predict the exciton bright-dark splitting but the results are highly dispersed with values in the range 10–40 meV and more importantly with different signs: depending on the methods applied, dark excitons lie above or below the dark ones^16–20^. It is therefore crucial to have a clear experimental determination.

Unfortunately, all attempts to measure the bright-dark splitting in MoS_2_ ML were not conclusive so far. The main reasons given were related to (i) the low optical quality of samples (without hBN encapsulation)^13^, (ii) the small value of the expected splitting compared to the luminescence/absorption linewidth or (iii) the low thermal population of the dark states if they lie above the bright ones^12^.

In this letter, we present the unambiguous optical emission spectrum of the dark exciton in MoS_2_ ML. In contrast to most predictions our measurements demonstrate that the spin-forbidden dark exciton lies below the bright exciton with a bright-dark splitting of +14 meV.

To this end, we performed magneto-photoluminescence experiments up to 30 T on MoS_2_ monolayers encapsulated in hexagonal Boron Nitride (hBN) with a magnetic field oriented along the monolayer plane (Voigt geometry). The high quality of the investigated samples allowed us to determine accurately the bright-dark energy splitting. We performed similar measurements in MoSe_2_ MLs encapsulated in hBN where a bright-dark splitting of −1.3 meV is determined in agreement with a recent report^21^. Measurements performed in a tilted magnetic field (45° with respect to the ML plane) confirm the spin-forbidden nature of the observed transitions and allow us to provide a conceivable description of the neutral exciton fine structure. The in-plane field component yields a brightening of the dark states and the out-of plane component induces a Zeeman spitting of these states yielding the first measurements of the dark exciton *g*-factor in MoS_2_ and MoSe_2_ monolayers. Remarkably, we find that the energy splitting between the bright and dark excitons in MoSe_2_ ML is very similar to the short range exchange energy which splits the two dark exciton states, a very original and unique situation for semiconductor nanostructures.

## Results

### Mixing of exciton states in magnetic field

**Fig. 1:**
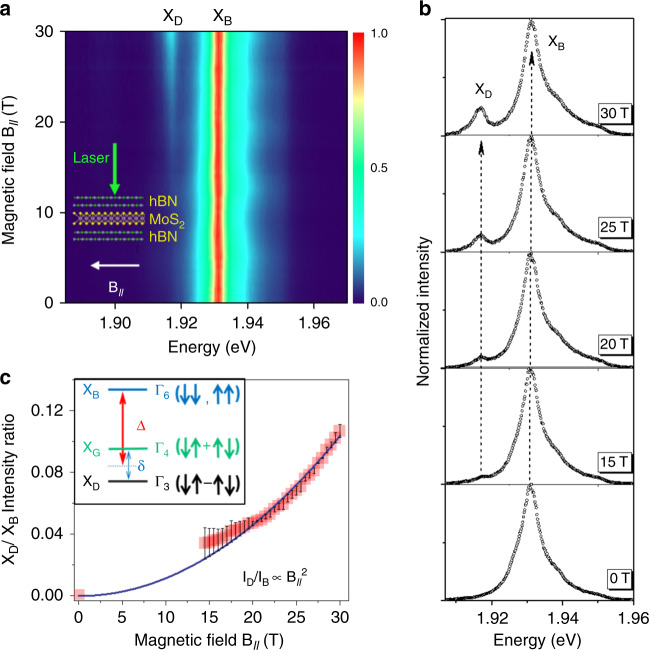
In-plane magnetic field B_//_—spin-forbidden dark exciton in MoS_2_ monolayer encapsulated in hBN revealed by photoluminescence. **a** Color map of the variation of the PL intensity as a function of **B**_**//**_ (the PL intensity of the bright exciton has been normalized at each field); **b** PL spectra for magnetic fields from 0 to 30 T showing the emergence of the brightened dark exciton at low energy. **c** Ratio of the PL intensity of dark (X_D_) and bright (X_B_) excitons as a function of magnetic field. Inset: sketch of the excitonic fine structure. The arrows ↑ and ↓ represent the main spin contribution of conduction and valence electrons involved in the exciton states (see ref. ^22^ for more details).

First, let us recall that the point symmetry group of a TMD monolayer is D_3h_. The direct band gap is located at the edges of hexagonal Brillouin zone, at the non-equivalent valleys *K*_±_. Because of the large spin–orbit splitting in the valence band, we restrict here our description to A excitons composed of an electron from one of the two conduction bands split by the spin–orbit interaction Δ_SO_ and a hole from the upper valence band A. We also consider only direct excitons (with a center of mass wave-vector *K* = 0). Then the exciton fine structure includes the two optically active (bright) excitons X_B_ with parallel spin ↑↑, ↓↓ (symmetry Γ_6_) and the two spin-forbidden dark states X_G_(↑↓ + ↑↓) and X_D_(↑↓–↑↓) (symmetry Γ_4_ and Γ_3_ respectively). The inset of Fig. 1c shows the bright-dark splitting Δ and the gray-dark splitting *δ* due to exciton exchange energy. It was shown that these dark states are optically forbidden for in-plane polarized light but that the X_G_ (Γ_4_) state can couple to out-of plane polarized light (Oz direction)^12,22,23^ and because of this is called here “gray” exciton.

An in-plane magnetic field **B**_**//**_ (Voigt geometry) mixes the spin components of the 2D exciton states^24,25^. For a TMD monolayer, this field interacts with the conduction band electron within the exciton and we can neglect its interaction with the hole because of the very large valence band spin–orbit splitting^13,14^. As a consequence the in-plane magnetic field brightens both X_G_ and X_D_ dark exciton states; the mixed bright-dark exciton states couple to in-plane polarized light allowing a straightforward determination of the energy of the dark states^13,14^. On the other hand, an out-of plane magnetic field **B**_**z**_ (Faraday geometry) leads to the Zeeman splitting of both bright and dark states and to the mixing between X_G_ and X_D_^22^. Thus, in tilted magnetic field, the four excitons are mixed and split so that it becomes possible to extract the *g*-factor of dark excitons. The (4 × 4) Hamiltonian describing the mixing between the four states under a given field with an angle *θ* with respect to the normal of the ML plane (*θ* = 90° for Voigt and *θ* = 0° for Faraday geometries) is described in the Supplementary Note 6.

### Magneto-photoluminescence for in-plane magnetic field

First we have investigated the effect of an in-plane magnetic field (Voigt configuration) on the low-temperature photoluminescence spectra in MoS_2_ monolayer. The 2D color map of PL intensity as a function of magnetic field from 0 to 30 T is plotted in Fig. 1a. In zero field, the emission is composed of a unique line corresponding to the radiative recombination of the bright exciton X_B_ at 1.931 eV in agreement with previous reports^26^. Remarkably we observe at low energy, typically 14 meV below X_B_, an additional peak which shows up above ~12 T. This feature, interpreted as the brightened spin-forbidden dark exciton, has been reproduced on several samples and spot positions (see data in Supplementary Note 1 principle), this line should correspond to both brightened gray and dark excitons, but the inhomogeneous linewidth in our samples is too broad (the linewidth of X_D_ is 5 meV) to enable us to distinguish the expected small splitting *δ* between gray and dark states. In a simple two-level system (*δ* = 0) where the in-plane magnetic field couples the bright and dark states, one expects that the ratio between the PL intensity of the bright and the dark PL lines follows a simple quadratic law: *I*_D_/*I*_B_ ~ (**B**_**//**_)^2^, where **B**_**//**_ is the amplitude of the in-plane magnetic field^14^. In Fig. 1c, we present the magnetic field dependence of the ratio between the PL intensity of the low-energy and the high-energy lines (corresponding to exciton states dominated by dark and bright components, respectively). The measured quadratic behavior is a strong indication that the low-energy line corresponds to the recombination of the dark excitons which have been brightened by the application of the magnetic field. An additional evidence for assigning the low-energy line observed for large in-plane magnetic field to spin-forbidden dark excitons is the measurements of its *g*-factor in an external field perpendicular to the monolayer. The measurements in the tilted field geometry presented below yield $g_z^D$ = −6.5; this large value is in good agreement with the predicted one in a simple model^27^ (see Supplementary Note 9) and the measured one in WSe_2_ MLs^22,28^ (Table 1).

**Table 1:** Measured exciton fine structure parameters for hBN-encapsulated TMD monolayers.

	MoS_2_	MoSe_2_	WSe_2_	WS_2_
Splitting between bright and dark exciton Δ (meV)	+14	−1.4 −1.5^21^	+40^12,15^	+55^12^ +40^42^
Splitting between gray and dark excitons *δ* (meV)	<2	<1	0.6^22,28^	0.5^42^
Bright exciton *g*-factor	−1.8	−4.0	−4.25^43^	−4.0^29^
Dark exciton *g*-factor	−6.5	−8.6	−9.4^22,28^	—
Transverse electron *g*-factor	2	2 2^21^	2^28^	—

**Fig. 2:**
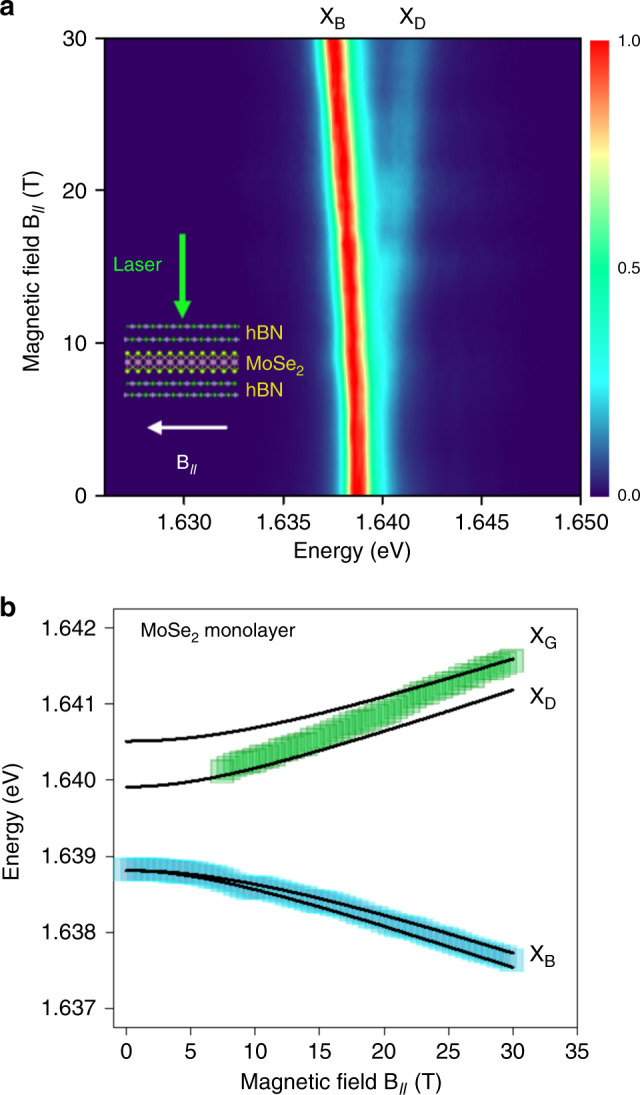
In-plane magnetic field B_//_—spin-forbidden dark exciton in MoSe_2_ monolayer encapsulated in hBN revealed by photoluminescence. **a** Color map of the variation of the PL intensity as a function of **B**_**//**_ (the PL intensity of the bright exciton has been normalized at each field); **b** energy of dark and bright excitons as a function of **B**_**//**_. The solid lines correspond to the fit described in the text.

We have also evidenced the energy of the spin-forbidden dark exciton in MoSe_2_ MLs encapsulated in hBN using the same experimental approach. As shown in the 2D color map of the PL spectra in Voigt configuration presented in Fig. 2a, the brightened dark exciton line (labeled for simplicity X_D_) lies above the bright exciton one (X_B_) in contrast to MoS_2_ (compare with Fig. 1a). Moreover, the brightened dark exciton starts to be visible at much lower field (~8 T) and the energy of the two lines vary much strongly with **B**_**//**_ as a consequence of the much smaller bright-dark splitting Δ. Again, the linewidth of the transitions (1.4 meV for X_B_ and 2.4 meV for X_D_) does not allow us to observe the splitting *δ* between gray and dark excitons. Using the general Hamiltonian presented in the Supplementary Note 6, including the effect of the exciton exchange interaction and the external magnetic field (with arbitrary orientation *θ* with respect to the ML plane), we can easily calculate the **B**_**//**_ field dependence of the bright, gray and dark exciton energies. Taking *θ* = 90° (Voigt geometry) and *δ* = 0.6 meV, we can fit our data (black solid lines in Fig. 2b) with Δ = −1.4 ± 0.1 meV and $g_{//}$ = 2.0 ± 0.2. These values are in good agreement with very similar measurements published very recently^21^. Note that the fit is very weakly sensitive to the value of *δ* for *δ* < 1 meV. Supplementary Figure 9a shows the results of the fit with three values of *δ* (0, 0.6, and 2 meV). In Fig. 2b, we present the results of the fit for *δ* = 0.6 meV which corresponds to the value experimentally measured in WSe_2_^22,28^. As *δ* is due to short range exchange interaction, which scales with the exciton binding energy, we do not expect the values to be strongly different between TMD materials as the exciton binding energies are roughly the same^29^.

In MoTe_2_ ML encapsulated with hBN, our measurements did not reveal any new PL line at high magnetic field that could have been attributed to spin-forbidden dark excitons. Our interpretation is that these excitons have a higher energy compared to the bright ones. Thus, a very small thermal population of dark states is expected and a much larger magnetic field is required to mix them with bright excitons. This is consistent with the fact that the calculated CB spin–orbit splitting is twice as large as for MoTe_2_ as for MoSe_2_^19^.

### Magneto-photoluminescence in tilted magnetic field

In order to have a full description of the exciton fine structure, we have measured the PL spectra of MoS_2_ and MoSe_2_ monolayers in a tilted magnetic field configuration (the field is oriented 45° with respect to the 2D layer plane). These experiments allow the determination of several key parameters, such as the dark exciton *g*-factors. In an oversimplified description, one can consider separately the effect of the two field components on the exciton spectra. The in-plane component of the field yields the mixing of the bright and dark exciton as already observed in the Voigt configuration and the out-of plane component leads to a Zeeman splitting of the states. While the energy splitting between the two spin/valley states for the bright exciton depends linearly with this *z*-component field (the slope given by effective *g*-factor), the field dependence of the dark states is more complicated since the two dark states at zero field are split by the exchange energy *δ* of the order of a few hundreds of µeV^22,28^ (inset of Fig. 1c).

**Fig. 3:**
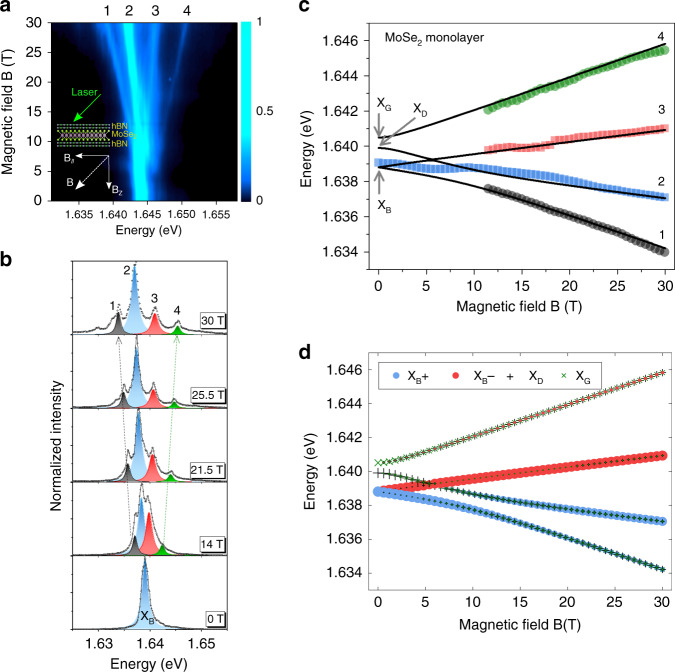
Tilted (45°) magnetic field—the four mixed excitons states (labeled 1, 2, 3, 4) in MoSe_2_ monolayer revealed by magneto-photoluminescence. **a** Color map of the variation of the PL intensity as a function of **B**; **b** PL spectra for magnetic fields from 0 to 30 T showing the emergence of the four mixed states (the intensity is normalized to the main exciton line (state 2)). **c** Magnetic field dependence of the energy of the four mixed exciton states. The full lines are fits to the model described in the text. The notations dark exciton (X_D_), gray exciton (X_G_) and bright excitons (X_B_) are only strictly valid at 0 T. **d** Same fitting results than in **c**. The size of each symbol is proportional to the weight of each component (the two bright component X_B_+ and X_B_− and the two dark component X_G_ and X_D_) (see Supplementary Note 7 for the calculation of the weight of the four components at high field).

First we present the dependence of the PL spectra of MoSe_2_ monolayer in tilted magnetic field. At high magnetic field (above ~12 T), the color map of the PL intensity in Fig. 3a and the PL spectra in Fig. 3b clearly evidence 4 lines corresponding to the 4 exciton states whose energy vary almost linearly with the field in the range 15–30 T (i.e., the out-of plane component varying from ~10 to 21 T). For lower magnetic field values, the energy splitting between the states is comparable to the PL linewidth preventing an accurate determination of the energy of the 4 states. However, we observe a clear non-linear dependence of the energy of the main PL line (which corresponds at *B* = 0 to the bright exciton) (Fig. 3c). This is a consequence of both the effect of the zero-field bright-dark splitting Δ and the zero-field splitting between the gray and dark states *δ*.

Thus, the rigorous description requires to consider both the exciton interaction with the tilted magnetic field **B** = **B**_**//**_+**B**_**z**_ and the exciton exchange interaction; **B**_**//**_ = Bsinθ and **B**_**z**_ = Bcosθ are the magnetic field components parallel and perpendicular to the monolayer plane (in the experiments of Fig. 3, *θ* = 45°). The interaction with the magnetic field is driven by $g_z^B$ and $g_z^D$, which are respectively the exciton *g*-factor of bright and dark excitons and $g_{//}$, which is the in-plane electron *g*-factor. The eigenstates for each magnetic field value have been obtained numerically (Supplementary Note 6). On the basis of this model and using the values Δ = −1.4 meV and $g_{//}$ = 2 obtained from the Voigt experiments (Fig. 2), one can fit simultaneously the field dependence of the energy of the 4 lines presented in Fig. 3c (see the solid line for the calculated curves). The agreement between the experiment and theory is very good. Interestingly, a clear anti-crossing is evidenced in the low field region as a consequence of the interplay of the transverse and longitudinal field components. This fitting procedure yields the *g*-factor of both the bright and dark states: we find $g_z^B$ = −4.0 and $g_z^D$ = −8.63. The value of the dark exciton *g*-factor around −9 is very similar to WSe_2_ ML^22,28^; it is an additional proof of the highlighting of the spin-forbidden dark states. As we cannot extract the energy of the 4 lines at weak magnetic field due to their linewidths, the fit is not very sensitive to the zero-field splitting *δ* between the gray and dark states (the curves have been calculated for *δ* = 0.6 meV, the value measured in WSe_2_ ML^22,28^). Supplementary Figure 9b shows the results of the fit with three values of *δ* (0, 0.6, and 2 meV). Note that the labeling of the 4 lines (X_B_, X_B_, X_D_, X_G_) in Fig. 3 is only valid at zero field. When **B** ≠ 0, the 4 states are mixed. In Fig. 3d, we show the mixing of each state as a function of **B**. In Supplementary Note 7, we show the calculation of the weight of the 4 components at 30 T.

**Fig. 4:**
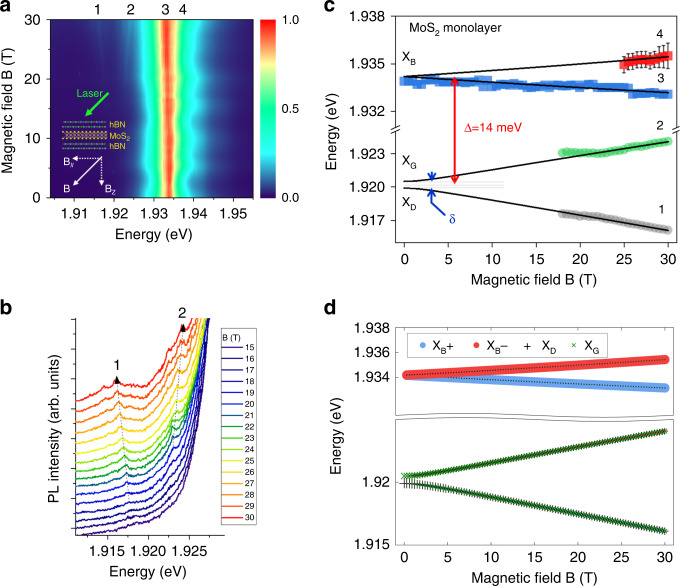
Tilted (45°) magnetic field—the four mixed excitons states (labeled 1, 2, 3, 4) in MoS_2_ monolayer revealed by magneto-photoluminescence. **a** Color map of the variation of the PL intensity as a function of B; **b** PL spectra for magnetic fields from 15 to 30 T showing the emergence of the two lowest energy states 1 and 2 (mainly with gray and dark components). **c** Magnetic field dependence of the energy of the four mixed exciton states. The full lines are fits to the model described in the text. The notations dark exciton (X_D_), gray exciton (X_G_) and bright excitons (X_B_) are only strictly valid at 0 T. **d** Same fitting results than in **c**. The size of each symbol is proportional to the weight of each component (the two bright component X_B_+ and X_B_− and the two dark component X_G_ and X_D_) (see Supplementary Note 7 for the calculation of the weight of the four components at high field).

Finally, we have measured the excitons spectra of MoS_2_ ML in tilted magnetic field. Due to the larger PL linewidth compared to the one of MoSe_2_ ML, it is more difficult to evidence the 4 excitons states as in Fig. 3a. However the energy of the two Zeeman split dark states can be extracted above 15 T as shown in Fig. 4a–c displays the measured magnetic field dependence of the energies of the four states together with the fit based on the same model as MoSe_2_. Using Δ = +14.0 meV and $g_{//}$ = 2 (determined previously in the Voigt geometry), the best fit is obtained for $g_z^B$ = −1.8 and $g_z^D$ = −6.5. The bright exciton *g*-factor of about −2 was already measured in high-quality MoS_2_ monolayer^30^. However, this is here the first measurement of the dark exciton *g*-factor. Interestingly, we note that the *g*-factors of both bright and dark excitons are significantly smaller in MoS_2_ than in other TMD materials. We can speculate that this is due to the very small conduction band spin–orbit splitting. Similarly, to MoSe_2_, the fit is not sensitive to the value of the dark-gray splitting due to the lack of experimental data at low field (the calculation has been done for *δ* = 0.6 meV). Supplementary Figure 9c shows the results of the fit with three values of *δ* (0, 0.6, and 2 meV). Table 1 summarizes the parameters of the exciton fine structure of MoS_2_ and MoSe_2_ monolayers presented in this Letter, together with the ones of WS_2_ and WSe_2_ MLs from previous work.

## Discussion

The measured value of Δ = 14 meV in MoS_2_ ML generates several remarks and interrogations.

(i) First it demonstrates the key role played by the exciton exchange energy contribution to the bright-dark energy splitting. The bright-dark exciton splitting Δ includes three contributions^17–19^:

Δ = Δ_SO_ + Δ_bind_ + Δ_exch._, where Δ_SO_ is the conduction band spin–orbit splitting, Δ_bind_ is the difference between the binding energies of bright and dark excitons due to the slightly different masses of spin ↑ and spin ↓ conduction bands and Δ_exch_ is the short range exciton exchange energy. Although the actual value of the spin–orbit splitting is not yet known, the most widely used calculated value in the literature is Δ_SO_ = −3 meV^31^. In a first approximation, the binding energy is proportional to the reduced mass of the exciton. Taking the calculated effective masses from ref. ^31^ and the experimental value of the bright exciton binding energy from ref. ^29^, we infer Δ_bind_ = +8 meV. As a consequence, our measurement of Δ = +14 meV demonstrates that the exciton exchange energy is crucial to determine the amplitude and the sign of the bright-dark energy splitting. Thus we deduce Δ_exch_ ~+9 meV (if indeed Δ_SO_ = −3 meV and Δ_bind_ = +8 meV). Note that the CB spin–orbit splitting in MoS_2_ monolayer has been recently estimated from transport measurements^32^; a splitting of about 15 meV was measured for an electron density of a few 10^12^ cm^−2^. This value five times larger than the calculated one includes significant band renormalization induced by many body effects.

(ii) Importantly, our measurements show that the splitting between spin-forbidden bright and dark excitons in MoS_2_ ML has an opposite sign compared to the calculated spin–orbit splitting in the conduction band. This could have important consequences for the trion fine structure^33^ .

(iii) Our measurements raise the question of the simple interpretation of MoS_2_ as a bright material. Indeed the TMD monolayers are usually divided into two categories: the so-called dark materials such as WS_2_ and WSe_2_ MLs, where the spin-forbidden dark excitons lie at lower energy compared to the bright ones. As a consequence, these monolayers are characterized by a rather weak luminescence yield at low temperature while the intensity increases with temperature due to thermal activation of bright states^6–9^. In contrast, MoX_2_ monolayer (X = S, Se, or Te) are often considered as “bright” materials as they exhibit stronger PL intensity at low temperature than their W-based counterparts but their intensity drops with temperature. This difference was assumed to vouch for dark excitons lying above the bright ones in MoX_2_. This bright-dark ordering has indeed been observed very recently for MoSe_2_ ML^21^ and confirmed by our results presented above. The measurements displayed in Fig. 1 show that the ordering is surprisingly opposite in MoS_2_ ML although the temperature dependence of the PL intensity in MoS_2_ ML is very similar to MoSe_2_ (strong at low temperature and decrease when temperature increases). Thus, we cannot use this simple argument to distinguish between a bright and a dark material as our results prove that the dependence of PL intensity with temperature in MoS_2_ may be the result of a complex relaxation scheme between bright and dark states as well as its interplay with non-radiative channels.

In addition, one can wonder why the gray exciton cannot be detected at zero magnetic field using high numerical aperture objective like in WS_2_ and WSe_2_^12^ (Supplementary Note 4). Remarkably, we note in Fig. 1 that the PL intensity of the mixed dark-bright state in transverse magnetic field is very weak (compared to similar experiments performed in WS_2_ or WSe_2_ MLs^12,13^) and that very high field are required to sizably observe it (larger than 14 T). We can speculate that either the oscillator strength of gray exciton is much smaller than in WX_2_ and/or that their population remains weak despite lying at lower energy than the bright state. We can tentatively explain the small oscillator strength by noticing that the smaller spin–orbit interaction in MoS_2_ compared to WS_2_ or WSe_2_ may yield a weaker oscillator strength of the gray exciton since it is related to the spin–orbit mixing with higher energy bands^12,19^. For the population issue, we can notice that contrary to WX_2_, the bright-dark exciton splitting in MoS_2_ ML is smaller than the optical phonon energies which can lead to inefficient relaxation between bright and dark excitons (the Γ_5_ phonon that permit relaxation from bright to dark exciton is 36 meV)^3,34^.

Another striking difference between TMD materials is the amplitude of spin/valley polarization for excitons. Although strong valley polarization and valley coherence have been measured in WSe_2_, WS_2_, and MoS_2_ MLs, the polarization in MoSe_2_ and MoTe_2_ MLs is very weak^35,36^ except under quasi-resonant excitation where significant polarization has been measured for the trion in MoSe_2_^37^. Recently, it was proposed that a crossing of bright and dark exciton dispersion curves combined with a Rashba effect associated with local fluctuations of electric field can lead to very fast spin relaxation^11^ . Our measurements of bright-dark splitting in MoS_2_ and MoSe_2_ are perfectly consistent with this scenario: the small negative splitting Δ = −1.4 meV in MoSe_2_ combined with a larger effective mass for dark excitons should lead to a crossing between bright and dark dispersions while the positive splitting Δ = +14 meV in MoS_2_ guarantees no crossing and as a consequence significant spin/valley exciton polarization measured in MoS_2_ MLs under CW optical orientation experiments^38–40^.

In conclusion, we have performed magneto-photoluminescence experiments in transverse and tilted magnetic fields up to 30 T in high-quality MoS_2_ and MoSe_2_ monolayers. These investigations yield the unambiguous determination of the bright-dark exciton splitting and the dark exciton *g*-factor. Such fundamental parameters are key elements to understand the optoelectronic and spin/valley properties of these 2D semiconductors as well as their associated van der Waals heterostructures.

## Methods

### Sample fabrication

We fabricated high-quality samples by encapsulating MoS_2_ and MoSe_2_ MLs in hexagonal boron nitride (hBN). The heterostructures are fabricated onto SiO_2_ (80 nm)/Si substratesusing a dry stamping technique^12,41^. The typical thickness of the top (bottom) hBN layer is ∼10 (200) nm and the typical in-plane size of the ML is ∼10 × 10 μm^2^.

### Experimental setup

Low-temperature magneto-PL experiments are performed in the Voigt configuration (magnetic field parallel to the layer plane) or tilted configuration (field oriented 45° with respect to the ML plane) using an optical fiber-based insert placed in a resistive solenoid producing magnetic fields up to 30 T. The samples are placed on top of an *x*–*y*–*z* piezo-stage kept in gaseous helium at *T* = 4.2 K. The light from a cw 515 nm laser is coupled to a mono-mode optical fiber with a core diameter of 5 μm and focused on the sample by an aspheric lens (spot diameter around 2 μm). The PL signal is collected by the same lens, injected into a multi-mode optical fiber of 50 µm core diameter, and analyzed by a 0.5 m long monochromator equipped with a charge-coupled device (CCD) camera. A sketch of the setup is shown in the Supplementary Methods.

### Reporting summary

Further information on research design is available in the Nature Research Reporting Summary linked to this article.

## Supplementary information

Supplementary Information

Peer Review File

Reporting Summary

## Data Availability

The data that support the findings of this study are available from the corresponding author upon request.

## References

[1] Wang G (2018). Colloquium: excitons in atomically thin transition metal dichalcogenides. Rev. Mod. Phys..

[2] Jones A (2013). Optical generation of excitonic valley coherence in monolayer WSe2, Nature. Nano.

[3] Dery H, Song Y (2015). Polarization analysis of excitons in monolayer and bilayer transition-metal dichalcogenides. Phys. Rev. B.

[4] Back P, Zeytinoglu S, Ijaz A, Kroner M, Imamoglu A (2018). Realization of an electrically tunable narrow-bandwidth atomically thin mirror using monolayer MoSe2. Phys. Rev. Lett..

[5] Koperski M (2017). Optical properties of atomically thin transition metal dichalcogenides: observations and puzzles. Nanophotonics.

[6] Wang G (2015). Spin-orbit engineering in transition metal dichalcogenide alloy monolayers. Nat. Commun..

[7] Zhang X-X, Yumeng Y, Zhao S, Yang F, Heinz T (2015). Experimental evidence for dark excitons in monolayer WSe2. Phys. Rev. Lett..

[8] Withers F (2015). WSe2 light-emitting tunneling transistors with enhanced brightness at room temperature. Nano Lett..

[9] Arora A (2015). Excitonic resonances in thin films of WSe2: from monolayer to bulk material. Nanoscale.

[10] Vinattieri A (1994). Exciton dynamics in GaAs quantum wells under resonant excitation. Phys. Rev. B.

[11] Yang M (2019). Exciton valley depolarization in monolayer transition-metal dichalcogenides. PRB.

[12] Wang G (2017). In-plane propagation of light in transition metal dichalcogenide monolayers: optical selection rules. Phys. Rev. Lett..

[13] Molas MR (2017). Brightening of dark excitons in monolayers of semiconducting transition metal dichalcogenides. 2D Mater..

[14] Zhang X (2017). Magnetic brightening and control of dark excitons in monolayer WSe2. Nat. Nano.

[15] Zhou Y (2017). Probing dark excitons in atomically thin semiconductors via near-field coupling to surface plasmon polaritons. Nat. Nano.

[16] Malic E (2018). Dark excitons in transition metal dichalcogenides. Phys. Rev. Mat..

[17] Deilmann T, Thygesen K (2017). Dark excitations in monolayer transition metal dichalcogenides. Phys. Rev. B.

[18] Qiu DY, Cao T, Louie SG (2015). Nonanalyticity, valley quantum phases, and lightlike exciton dispersion in monolayer transition metal dichalcogenides: theory and first-principles calculations. Phys. Rev. Lett..

[19] Echeverry JP, Urbaszek B, Amand T, Marie X, Gerber IC (2016). Splitting between bright and dark excitons in transition metal dichalcogenide monolayers. Phys. Rev. B.

[20] Peng G (2019). Distinctive signatures of the spin- and momentum-forbidden dark exciton states in the photoluminescence of strained WSe 2 monolayers under thermalization. Nanoletters.

[21] Lu Z (2020). Magnetic field mixing and splitting of bright and dark excitons in monolayer MoSe2. 2D Mater..

[22] Robert C (2017). Fine structure and lifetime of dark excitons in transition metal dichalcogenide monolayers. Phys. Rev. B.

[23] Slobodeniuk AO, Basko DM (2016). Spin–flip processes and radiative decay of dark intravalley excitons in transition metal dichalcogenide monolayers. 2D Mater..

[24] Amand T (1997). Spin quantum beats of 2D excitons. Phys. Rev. Lett..

[25] Mashkov IV, Gourdon C, Lavallard P, Yu Roditchev D (1997). Exciton quantum beats in type-II GaAs/AlAs superlattices in longitudinal and in-plane magnetic fields. Phys. Rev. B.

[26] Robert C (2018). Optical spectroscopy of excited exciton states in MoS2 monolayers in van der Waals heterostructures. Phys. Rev. Mat..

[27] Koperski M (2019). Orbital, spin and valley contributions to Zeeman splitting of excitonic resonances in MoSe2, WSe2 and WS2 Monolayers. 2D Mater..

[28] Molas MR (2019). Probing and manipulating valley coherence of dark excitons in monolayer WSe2. Phys. Rev. Lett..

[29] Goryca M (2019). Revealing exciton masses and dielectric properties of monolayer semiconductors with high magnetic fields. Nat. Com..

[30] Cadiz F (2017). Excitonic linewidth approaching the homogeneous limit in MoS2-based van der Waals heterostructures. Phys. Rev. X.

[31] Kormanyos A (2015). k.p theory for two-dimensional transition metal dichalcogenide semiconductors. 2D Mater..

[32] Pisoni R (2018). Interactions and magnetotransport through spin-valley coupled Landau levels in monolayer MoS2. Phys. Rev. Lett..

[33] Roch J (2019). Spin-polarized electrons in monolayer MoS2. Nat. Nano.

[34] Jin Z, Li X, Mullen JT, Kim KW (2014). Intrinsic transport properties of electrons and holes in monolayer transition-metal dichalcogenides. Phys. Rev. B.

[35] Wang G (2015). Polarization and time-resolved photoluminescence spectroscopy of excitons in MoSe2 monolayers. APL.

[36] Robert C (2016). Excitonic properties of semiconducting monolayer and bilayer MoTe2. Phys. Rev. B.

[37] Tornatzky H, Kaulitz A-M, Maultzsch J (2018). Resonance profiles of valley polarization in single-layer MoS2 and MoSe2. Phys. Rev. Lett..

[38] Sallen G (2012). Robust optical emission polarization in MoS2 monolayers through selective valley excitation. Phys. Rev. B.

[39] Cao T (2012). Valley-selective circular dichroism of monolayer molybdenum disulphide. Nat. Commun..

[40] Mak K, He K, Shan J, Heinz T (2012). Control of valley polarization in monolayer MoS2 by optical helicity. Nat. Nano..

[41] Fang HH (2019). Control of the exciton radiative lifetime in van der Waals heterostructures. Phys. Rev. Lett..

[42] Zinkiewicz, M. et al. Neutral and charged dark excitons in monolayer WS2. Preprint at https://arxiv.org/abs/2005.14071 (2020). 10.1039/d0nr04243a 32853305

[43] Stier AV (2018). Magnetooptics of exciton Rydberg states in a monolayer semiconductor. Phys. Rev. Lett..

