# Supplementary material for: Measurement of the spin-forbidden dark excitons in MoS2 and MoSe2 monolayers
Source: Nat Commun. 2020 Aug 12;11:4037. doi: 10.1038/s41467-020-17608-4 (PMC7423942; doi:10.1038/s41467-020-17608-4)
Supplement: Supplementary file 1 — Supplementary Information [file 41467_2020_17608_MOESM1_ESM.pdf]

Measurement of the Spin-Forbidden Dark Excitons in MoS_2 and $MoSe_2$ monolayers

C. Robert, B. Han, P. Kapuscinski, A. Delhomme, C. Faugeras, T. Amand, M.R. Molas, M. Bartos, K. Watanabe, T. Taniguchi, B. Urbaszek, M. Potemski & X. Marie

SUPPLEMENTARY INFORMATION

Supplementary Methods

Experimental set-up

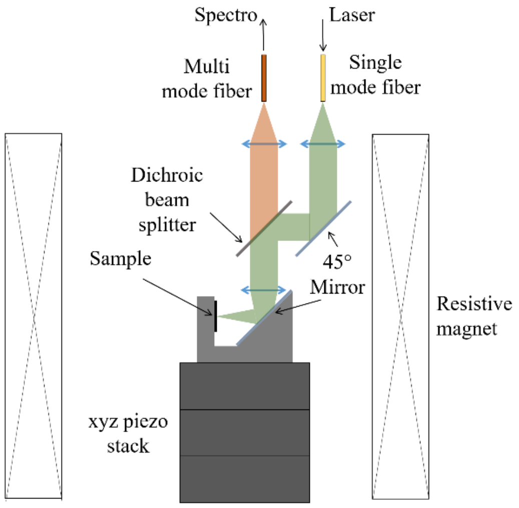

Supplementary Figure 1: Sketch of the optical setup in Voigt geometry. For tilted field experiments, the sample is directly glued on the bottom 45° mirror.

Supplementary Note 1: *Additional data in MoS_2*

The results presented in Fig.1 (brightening of the dark exciton in MoS_2 in Voigt geometry) have been reproduced in several samples and always give a bright-dark exciton splitting of +14 meV.

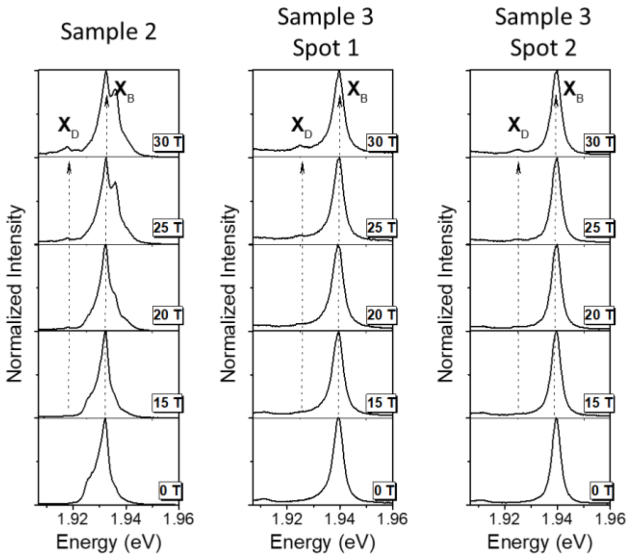

Supplementary Figure 2 : PL spectra for other MoS_2 samples than the one presented in the main text for in plane magnetic fields from 0 to 30 T showing the emergence of the brightened dark exciton X_D 14 meV below the bright exciton X_B.

Supplementary Note 2: *Excitation power dependence of the dark exciton*

We checked that the optical transition we interpreted as the dark exciton of MoS_2 has a PL intensity that varies linearly with excitation power. We can thus safely exclude that it originates from a defect state.

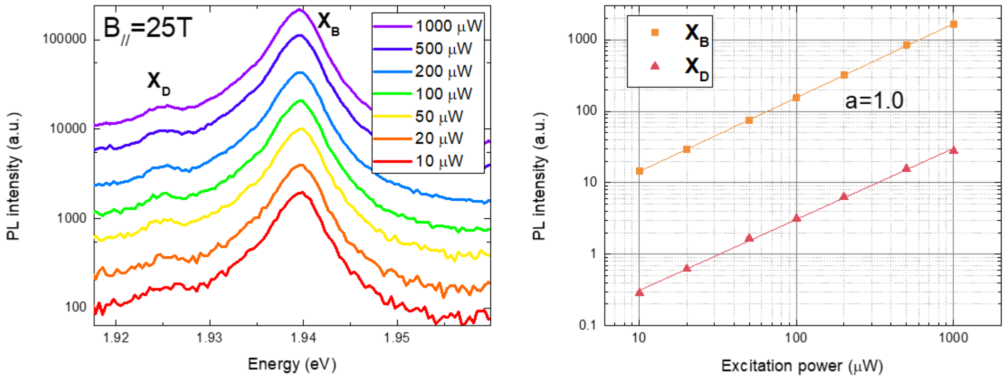

Supplementary Figure 3 : (Left) Normalized PL spectra for MoS_2 samples for in plane magnetic field of 25T as a function of excitation power. The spectra are shifted for clarity. (Right) Integrated intensity of bright and dark exciton lines at 25T as a function of power.

Supplementary Note 3: *PL spectra in wider spectral range*

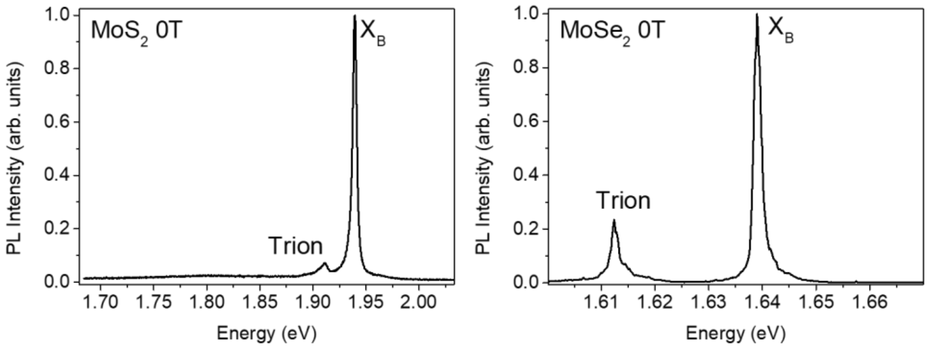

Supplementary Figure 4 : PL spectra of MoS2 and MoSe2 samples at 0T showing other exciton complexes, mainly the trion.

Supplementary Note 4: *Detection of out-of-plane polarized transition (grey exciton) in MoS2 using high numerical aperture objective*

We tried to detect the grey exciton X_G (z-polarized) in MoS_2 at zero magnetic by using a high numerical aperture objective (NA=0.82). This technique has proved to be very efficient to detect X_G in WSe2[1]. To distinguish between in-plane and out-of-plane transitions we imaged the Fourier plane of the objective and put a pinhole in that plane. The setup is exactly the same than in our previous work[1] and is sketched in Supplementary Figure 5(a). By translating this pinhole we can choose to detect light propagating perpendicularly to the ML (θ=0°) and detect only bright excitons or to detect light with an in-plane component (maximum angle for a NA=0.82 objective is θ=55°) to detect both grey excitons and bright excitons. The PL spectra of MoS2 in a logarithmic scale at 4 K for the two positions of the pinhole are shown in Supplementary Figure 5(b). We did not detect any signature of the grey exciton which should rise as a feature 14 meV below X_B in the red curve.

(a) (b)

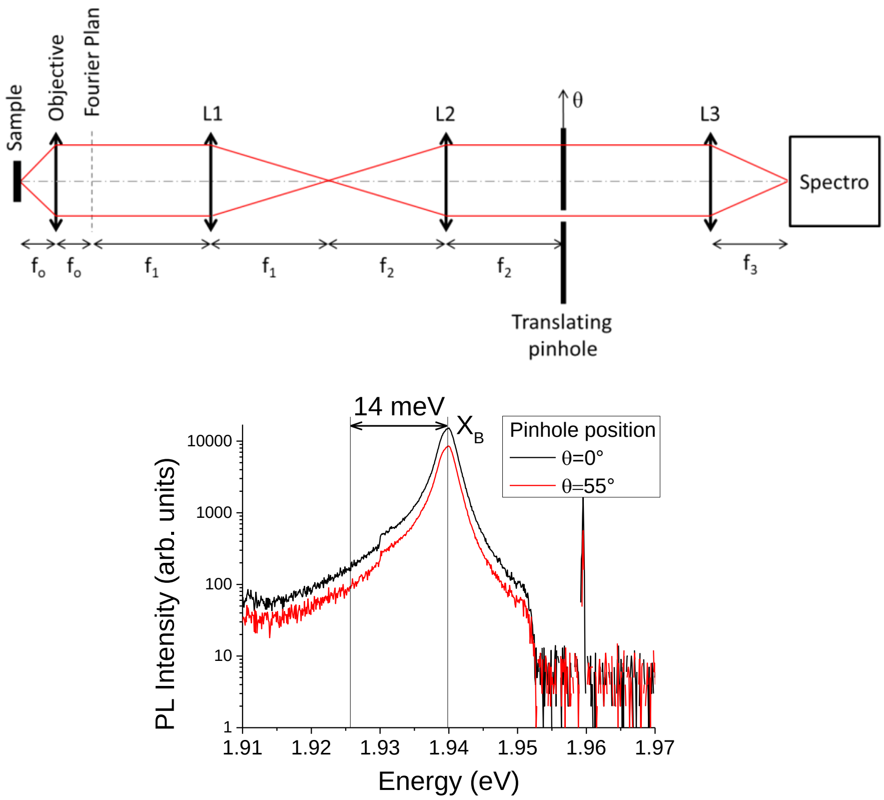

Supplementary Figure 5 : (a) Sketch of the optical setup enabling filtering of detected angles θ. (b) PL spectra of MoS_2 in a logarithmic scale at 4 K for the two positions of the pinhole.

Supplementary Note 5: ***Additional data in $MoSe_2$***

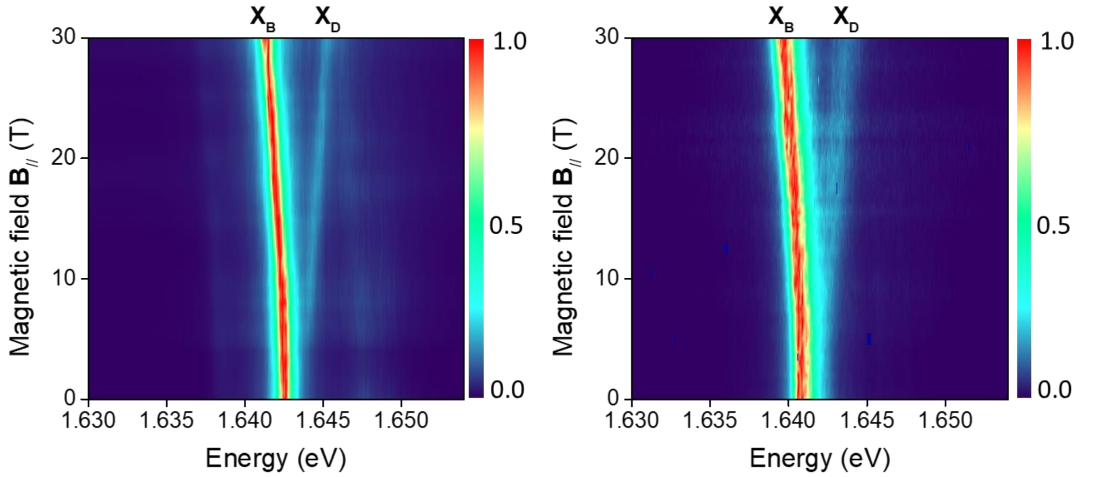

Figure SI6 : PL spectra for $MoSe_2$ samples for in plane magnetic fields from 0 to 30 T at two different spot positions than the one presented in the main text. The PL intensity of the bright exciton has been normalized at each field.

Supplementary Note 6: *Exciton fine structure in tilted magnetic field*

The TMD materials such as WSe_2, WS_2, MoS_2, $MoSe_2$ and MoTe2 crystallize in hexagonal structure which is invariant under the symmetry operations of the D_{3h} group[2]. In such materials, the conduction bands Bloch amplitudes U_m^γ in non-equivalent $K_\pm$ valleys belong to the D_{3h} representations: $\{\Gamma_8, \Gamma_9\}$, the latter being split by spin-orbit interaction, while the topmost valence bands (A) ones belong to Γ_7 representation[3]. The electron-hole Bloch-amplitude pair functions built with these states belong thus to the representation product $\Gamma_9 \otimes \Gamma_7^* \otimes \Gamma_{env}$ and $\Gamma_8 \otimes \Gamma_7^* \otimes \Gamma_{env}$ in notations of ref.[4] . Restricting ourselves to 1s exciton ground states, we have $\Gamma_{env} = \Gamma_1$, so that, all the single particle representations being two-dimensional, we obtain four exciton states in the A series with symmetry $\Gamma_9 \otimes \Gamma_7^* = \Gamma_5 + \Gamma_6$ and four others with symmetry $\Gamma_8 \otimes \Gamma_7^* = \Gamma_3 + \Gamma_4 + \Gamma_6$. However, using coupling tables, it can be seen that only the Γ_6 - excitons of $\Gamma_9 \otimes \Gamma_7^*$ and Γ_3 - and Γ_4 - excitons of $\Gamma_8 \otimes \Gamma_7^*$ are direct excitons, the Γ_6 doublet and Γ_4 singlet excitons being only coupled to light (dipole allowed). Defining the hole Bloch amplitude by $U_{\mp 1/2}^{h,7} = \pm \hat{K}\left(U_{\pm 1/2}^{7}\right)$, $\hat{K}$ being the time-reversal operator, these excitons Bloch amplitudes are denoted here by :

$$\Psi_{\pm 1}^6 = U_{\mp 1/2}^9 \otimes U_{\mp 1/2}^{h,7}, \tag{1}$$

coupled to $\sigma^\pm$ photons respectively, and

$$\begin{aligned} \Psi^4 &= \frac{i}{\sqrt{2}}\left(U_{+1/2}^8 \otimes U_{-1/2}^{h,7} + U_{-1/2}^8 \otimes U_{+1/2}^{h,7}\right) \\ \Psi^3 &= \frac{1}{\sqrt{2}}\left(U_{+1/2}^8 \otimes U_{-1/2}^{h,7} - U_{-1/2}^8 \otimes U_{+1/2}^{h,7}\right) \end{aligned} \tag{2}$$

Ψ^4 being the only one coupled to π^z photons (the Z-exciton mode[2]).

i. Coulomb exchange and spin-orbit interaction.

Beside direct Coulomb interaction, these excitons experience electron-hole exchange, so that, in the direct exciton manifold with basis $\mathrm{B} = \left\{\Psi_{+1}^6, \Psi_{-1}^6, \Psi^3, \Psi^4\right\}$ the effective exciton Hamiltonian, taking into account exchange and spin-orbit interaction[5], can be written as:

$$\mathrm{H}_{exch} = \frac{1}{2}\begin{pmatrix} \Delta & 0 & 0 & 0 \\ 0 & \Delta & 0 & 0 \\ 0 & 0 & -\Delta - \delta & 0 \\ 0 & 0 & 0 & -\Delta + \delta \end{pmatrix} \tag{3}$$

We turn now to the action of an external magnetic field **B** applied to the sample in any direction, and derive the corresponding Zeeman effective Hamiltonian.

We write its components in spherical coordinates as:

$$\mathbf{B} = \left(B\sin\theta\cos\varphi, \quad B\sin\theta\sin\varphi, \quad B\cos\theta\right)^T .$$

ii. Longitudinal part of the magnetic field: $B_z\mathbf{e_z}=B\cos\theta\mathbf{e_z}$

This component belongs to Γ_2 representation of D_{3h}. Thus, a pure longitudinal magnetic field splits the states Ψ^6_{+1} Ψ^6_{-1}, and couples the states Ψ^3 and Ψ^4. Using the coupling tables of ref.4, we have shown that this Hamiltonian can be presented in the form :

$$\mathrm{H}_{Bz} = \frac{1}{2}\mu_B B\cos(\theta)\begin{pmatrix} g_z^B & 0 & 0 & 0 \\ 0 & -g_z^B & 0 & 0 \\ 0 & 0 & 0 & ig_z^D \\ 0 & 0 & -ig_z^D & 0 \end{pmatrix} \quad (4)$$

iii. Transverse part of the magnetic field: $\boldsymbol{B}_{//}=B\sin\theta\cos\varphi\boldsymbol{e}_x + B\sin\theta\sin\varphi\boldsymbol{e}_y$

We have $B_x \pm iB_y = B\sin\theta e^{\pm i\varphi}$ which transforms like the spin operators $\hat{S}_x \pm i\hat{S}_y$. These components and operators belong to Γ_5 two-dimensional representation of D_{3h}. The product of exciton states representations:

$\Gamma_6^* \otimes \Gamma_6 = \Gamma_6 \otimes \Gamma_6 = \Gamma_1 + \Gamma_2 + \Gamma_6$, $\Gamma_3^* \otimes \Gamma_3 = \Gamma_3 \otimes \Gamma_3 = \Gamma_1 = \Gamma_4^* \otimes \Gamma_4$, $\Gamma_3^* \otimes \Gamma_4 = \Gamma_3 \otimes \Gamma_4 = \Gamma_2$, do not contain Γ_5 representation, but $\Gamma_6^* \otimes (\Gamma_3 + \Gamma_4) = 2\Gamma_5$. So it can be deduced that the transverse field only couples the Ψ^3 and $\Psi^6_{\pm1}$ excitons one hand, and the Ψ^4 and $\Psi^6_{\pm1}$ excitons, with *a priori* two different coupling constants. The Zeeman Hamiltonian contribution of the transverse field can thus be presented under the form:

$$\mathrm{H}_{B//} = \frac{1}{2\sqrt{2}}\mu_B B\sin(\theta)\begin{pmatrix} 0 & 0 & g_{//}e^{-i\varphi} & ig_{//}e^{-i\varphi} \\ 0 & 0 & -g_{//}e^{+i\varphi} & ig_{//}e^{+i\varphi} \\ g_{//}e^{+i\varphi} & -g_{//}e^{-i\varphi} & 0 & 0 \\ -ig_{//}e^{+i\varphi} & -ig_{//}e^{-i\varphi} & 0 & 0 \end{pmatrix} \quad (5)$$

, where we have assumed for simplicity that the two coupling constants are opposite. This can be justified by the fact that only the conduction electrons contribute significantly to the coupling.

The total Hamiltonian is thus, in the general case:

$$\boxed{H^X = H^X_{exch} + H^X_{B_z} + H^X_{B_{//}}} \quad (6)$$

We have checked that this Hamiltonian is strictly equivalent to the one written in ref.[6] expressed in a different basis fabricated with states in each valley. Indeed, the secular equation of the Eigen-equation of the system is independent of the basis choice and is given by:

$$[(\Delta - 2\lambda)^2 - (g_z^b\mu_B B\sin\theta)^2][(\Delta + 2\lambda)^2 - (g_z^d\mu_B B\cos\theta)^2 - \delta^2] +$$
$$+2(g_{//}\mu_B B\sin\theta)^2(\Delta^2 - 4\lambda^2 + g_z^b g_z^d\cos^2\theta) + (g_{//}\mu_B B\cos\theta)^4 = 0$$

As expected, it is clearly seen that the four Eigen-energies λ do not depend on the azimuthal angle φ of the magnetic field.
Note that this equation is also invariant when changing $g_{//}$ by $-g_{//}$, and by changing both signs of g_z^b and g_z^d, which shows that the sign of these two g-factors are linked. In the Hamiltonian (4) the bright g factor $g_z^b$is defined with the usual convention $g_z^b \mu_B B_Z = E_{\Psi_{+1}^6} - E_{\Psi_{-1}^6}$so that with $g_z^b < 0$ the energy of the bright exciton with the electron in K+ ($|\Psi_{+1}^6\rangle$ state) decreases when B_Z increases. The definition of the dark g factor g_z^d is not so obvious in the Hamiltonian (4). To explain our convention we can write the two dark states in each valley:

$$|K_+^d> \; = U_{+1/2}^8 \otimes U_{-1/2}^{h,7} = \frac{|\Psi^3> -i|\Psi^4>}{\sqrt{2}}$$

$$|K_-^d> \; = U_{-1/2}^8 \otimes U_{+1/2}^{h,7} = -\frac{|\Psi^3> +i|\Psi^4>}{\sqrt{2}}$$

In the Hamiltonian (S4), g_z^d is defined so that with $g_z^d < 0$ the energy of the dark exciton with the electron in K+ ($|K_+^d\rangle$ state) decreases when B_Z increases.
Thanks to the fitting of Fig. 3c, we experimentally confirmed that both g_z^b and g_z^d are indeed negative in $MoSe_2$ with our conventions.

Supplementary Note 7: *Calculation of the weight of each component in the mixed exciton states in tilted field at 30 T*

The solid lines in Fig.3c and Fig.4 are obtained by numerically calculate the eigenvalues of Hamiltonian (6). By calculating the eigenvectors we can determine the weight of each component ($\Psi^6_{+1}, \Psi^6_{-1}, \Psi^3, \Psi^4$) in the exciton states at 30 T. The results presented in the tables below for $MoSe_2$ and MoS_2 show that the states are strongly mixed.

**Calculated eigenvalues**	**1.6342 eV**	**1.6371 eV**	**1.6409 eV**	**1.6458 eV**
Ψ^6_{+1}	0.2479	0.7521	0	0
Ψ^6_{-1}	0	0	0.9325	0.0675
Ψ^3	0.3964	0.1331	0.0302	0.4402
Ψ^4	0.3557	0.1148	0.0373	0.4923

Supplementary Table 1: Weight in module of the 4 components ($\Psi^6_{+1}, \Psi^6_{-1}, \Psi^3, \Psi^4$ *) in the 4 eigenvalues of* $MoSe_2$ *calculated at 30 T for* θ*=45°. The main component is shown in grey.*

**Calculated eigenvalues**	**1.9161 eV**	**1.9241 eV**	**1.9332 eV**	**1.9354 eV**
Ψ^6_{+1}	0.0052	0	0.9948	0
Ψ^6_{-1}	0	0.0118	0	0.9882
Ψ^3	0.5346	0.4572	0.0024	0.0057
Ψ^4	0.4602	0.5310	0.0028	0.0061

Supplementary Table 2: Weight in module of the 4 components ($\Psi^6_{+1}, \Psi^6_{-1}, \Psi^3, \Psi^4$ *) in the 4 eigenvalues of* MoS_2 *calculated at 30 T for* θ*=45°. The main component is shown in grey.*

Supplementary Note 8: *Influence of δ on the fit of the energies of bright and dark states*

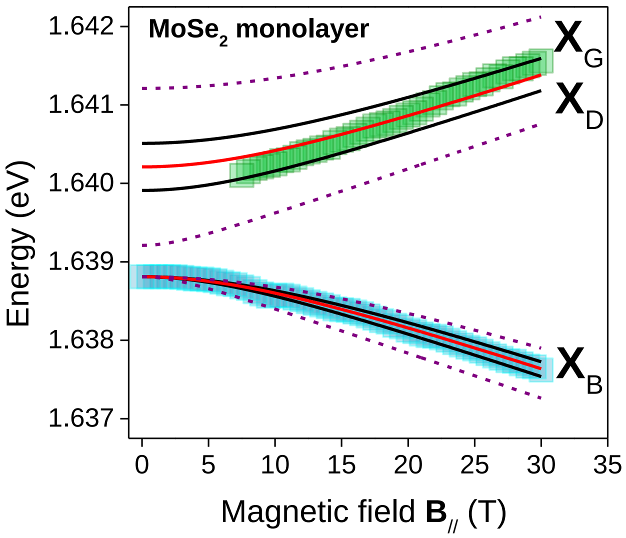

Supplementary Figure 9a : Energy of dark and bright excitons in $MoSe_2$ as a function of B//. Red, black and purple dashed solid lines correspond to the fit with δ=0 (2-band model), δ=0.6 meV (same as main text) and δ=2 meV.

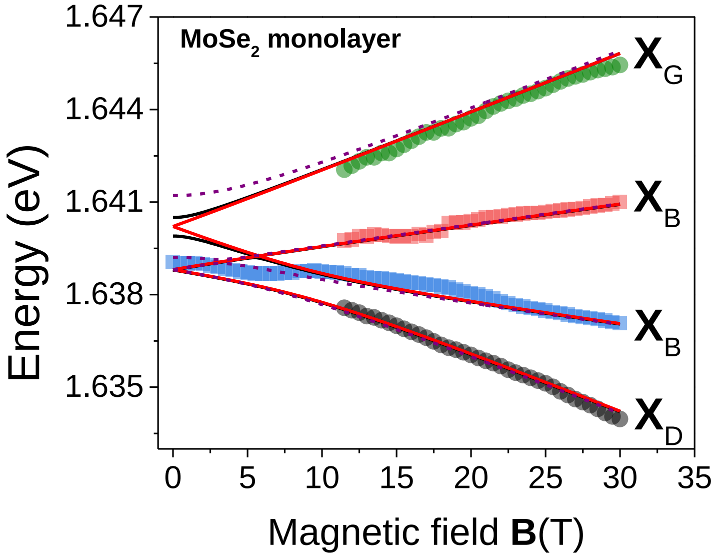

Supplementary Figure 9b : Energy of dark and bright excitons in $MoSe_2$ as a function of tilted (45°) field. Red, black and purple dashed solid lines correspond to the fit with δ=0 (2-band model), δ=0.6 meV (same as main text) and δ=2 meV.

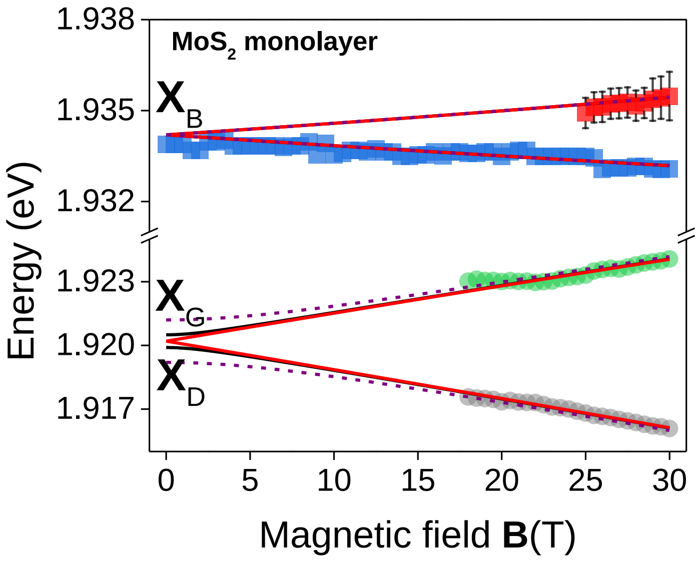

Supplementary Figure 9c : Energy of dark and bright excitons in MoS_2 as a function of tilted (45°) field. Red, black and purple dashed solid lines correspond to the fit with δ=0 (2-band model), δ=0.6 meV (same as main text) and δ=2 meV.

Supplementary Note 9: *Interpretation of the g factors in MoS2*

We can interpret the values of g factors of both bright and dark excitons based on a simple model separating the contributions of spin, valley and orbital in the value of the g factor. If we take into account only the spin and the orbital contributions we should get g_{XB}=-4 and g_{XD}=-8 which are roughly the values obtained for WSe2. In MoS2 the valley contribution seems to be more important. Taking the experimental value of g_{XB}=-1.8 we get a valley contribution of 2.2. If we assume that the valley contribution is similar for g_{XD} we expect that g_{XD}=-5.8. Of course taking the same valley contribution for both bright and dark excitons is a very rough assumption. In particular, as the conduction band masses involved in these two states is different, we could expect that the valley contribution for the dark exciton is smaller (larger conduction band mass). Nevertheless, we want to recall that this model is oversimplified and that theoretically predicting the g factor in semiconductors require much more sophisticated approach (see for instance recent DFT calculations[7,8]).

Supplementary References

[1] G. Wang *et al.*, PRL **119**, 047401 (2017)
[2] C. Robert *et al.*, Phys. Rev. B **96**, 155423 (2017)
[3] A. Kormanyos *et al*, 2D Mater. **2,** 022001 (2015)
[4] G. F. Koster, R. G.Wheeler, J. O. Dimmock, and H. Statz, Properties of the Thirty-Two Point Groups (MIT Press, Cambridge, Massachusetts, 1963).
[5] J. P. Etcheverry et al. Phys. Rev. B **93**, 121107(R) (2016)
[6] M.R. Molas et al, Phys. Rev. Lett. **123**, 096803 (2019)
[7] T. Deilmann, P. Krüger, and M. Rohlfing, Phys. Rev. Lett. **124**, 226402 (2020)
[8] J. Förste, N. V. Tepliakov, S. Y. Kruchinin, J. Lindlau, V. Funk, M. Förg, K. Watanabe, T. Taniguchi, A. S. Baimuratov, and A. Högele, ArXiv:2002.11646 [Cond-Mat] (2020)
